# Modification of a flexoelectric perovskite type LaMnO_3_ with APg-C_3_N_4_ for the removal of organic micropollutants: a synergy between tribo-catalysis and photo-illumination

**DOI:** 10.1186/s13065-025-01578-z

**Published:** 2025-07-04

**Authors:** Ajibola Abiodun Bayode, Andrea Osti, Antonella Glisenti

**Affiliations:** 1 Department of Chemical Sciences, Faculty of Natural Sciences, Redeemer’s University, P.M.B. 230, Ede, 232101 Nigeria; 2 Department of Chemical Sciences, University of Padova, Via F. Marzolo, 1, 35131 Padua, Italy

**Keywords:** Tribocatalysis, Tribo-photocatalysis, Rhodamine blue, Flexoelectricity, Methylene blue

## Abstract

Investigating the interaction between tribocatalysis and photocatalysis using the flexoelectric catalyst shows promising potential for water treatment. However, there is a lack of research on the combined effects of photocatalysis and tribocatalysis, especially when utilizing the flexoelectric catalyst. In this research, we synthesized the La_0.8_MnO_3_ modified with acid-activated porous Graphitic carbon nitride (APCNS) (La_0.8_MnO_3_@APCNS). We examined it using various methods such as Fourier transform infrared spectroscopy (FTIR), X-ray diffraction (XRD), and scanning electron microscopy (SEM). The tribocatalytic and tribo-photocatalytic performance of the La_0.8_MnO_3_@APCNS for degrading Rhodamine blue (RhB) and Methylene blue (MB) was explored. The results showed the PTFE magnetic bar, grinding wheel and La_0.8_MnO_3_@APCNS achieved degradation rates of 83.33% for RhB and 84.78% for MB within 360 min. When combining tribocatalysis and photocatalysis, the efficiency increased to 100% for RhB and 95.5% for MB within 90 min. Experimental evidence confirms the significant synergistic effects of combining both techniques. The scavenger test revealed that hydroxyl and superoxide radicals played a major role in degrading both dyes. The performance of the La_0.8_MnO_3_@APCNS remained consistent through the fifth cycle, for both dyes. This demonstrates effectiveness and underscores the potential practical and environmentally friendly aspects of tribo-photocatalytic techniques for purifying water.

## Clinical trial

Not applicable.

## Introduction

The swift increase in industrialization and globalization has notably improved our standard of living [1, 2]. However, many industries, such as food, electroplating, printing, and textiles, utilize synthetic dyes, leading to various forms of pollution such as air, water, and land pollution. The release of untreated dye wastewater into water bodies poses severe risks to both human health and the environment [3]. This is due to the highly complex structure of the dye wastewater due to its high organic concentration, thereby making degradation difficult.

As a result, there is a need to develop environmentally friendly, sustainable, and effective dye treatment methods. Wastewater treatment is crucial for the advancement of human society, and various treatment technologies have been explored, including adsorption [2], catalysis [4, 5], biodegradation [6], photocatalysis [7, 8], sonocatalysis [9], electrocatalysis [10, 11], Fenton process [12], ozonation [13] and hybrid microbial degradation [14]. However, these approaches have limitations, such as selective adsorption without decomposition, secondary pollution from chemical precipitation, high energy consumption in electrochemical methods, and lengthy degradation cycles in biodegradation [15–17]. Additionally, photocatalysis suffers from low solar energy utilization rates and carrier recombination [15]. Therefore, it is imperative to continue the search for more effective and environmentally benign wastewater treatment methods to solve the issue.

Friction energy, in addition to light energy, is also a plentiful and relatively steady source of renewable energy [18]. This energy can be generated from wind, water, or waves. The harvesting and usage of friction energy have gained significant attention recently due to its status as a clean energy source [19, 20]. The advancement of scientific knowledge has led to the discovery of harnessing mechanical/frictional energy to trigger the triboelectric effect, which can play a significant role in environmental remediation works [21], This is made possible using a newly developed catalytic technology called tribocatalysis [21, 22]. The principle of tribo-catalysis is based on the utilization of surrounding mechanical energy to drive electrochemical reaction degradation [23]. It allows self-power purification by enabling chemical processes to capture small amounts of mechanical energy from the surrounding environment, such as water movement and vibrations [18, 24]. Tribocatalysts can be made from several materials, both centrosymmetric and non-centrosymmetric like semiconductor catalysts, polytetrafluoroethylene (PTFE), fluorinated ethylene propylene (FEP), and other materials [25–27].

In recent studies, researchers have reported on tribo-catalysis tailored for non-centrosymmetric materials, with a focus on the influence of piezocatalysis involved in the process [16, 25, 28]. Specifically, in the perovskite structure LaMnO_3_, Mn ions are octahedrally coordinated by oxygen atoms, leading to the crystal field splitting of Mn 3d orbitals into t_2g_ and e_g_ orbitals [29]. LaMnO_3_, a non-centrosymmetric perovskite oxide with the space group R̅3c rhombohedral distortion from the ideal cubic perovskite structure), due to the Jahn-Teller effects considered an ideal candidate [30]. LaMnO_3_ is a ferromagnetic material with flexoelectric properties, excellent chemical stability, and a narrow band gap (E_g_ = 1.94 eV), making it suitable for photocatalysis [30]. Dhinesh et al. reported that LaMnO_3_, synthesized via the hydrothermal method, can degrade Methyl Orange (MO) solution over 60 min [31]. In another study, Priyatharshni et al. reported the complete decolouration of methylene violet dye (MV) up to ∼95% in 315 min through photocatalysis using LaMnO_3_ modified with Urea and CTAB [32].

Due to its strong photocatalysis performance, LaMnO_3_ is also believed to exhibit excellent tribo-catalysis performance for enhancing dye decomposition. To the best of our knowledge, perovskite oxide flexoelectric LaMnO_3_ has not been utilized in tribo-catalysis. Tribo-catalysis has the potential for application in the treatment of dye wastewater by utilizing environmental friction energy [30]. The main challenge in the tribocatalysis process is the extended duration required for degradation. To tackle this problem, a combination of photo-illumination and tribo-catalysis will result in an increased generation of electrons and holes, significantly decreasing the time needed for the degradation of a specific quantity of organic materials was examined.

This work investigates the tribocatalytic and tribophotocatalytic performance of a P-type 0D LaMnO_3_ perovskite oxide modified with an n-type 2D acid-activated graphitic carbon nitride (APCNS), forming a p-n heterojunction La_0_._8_MnO_3_@APCNS for the degradation of rhodamine blue (RhB) and methylene blue (MB) through a green approach which involves collection of mechanical friction energy harnessed by simple stirring. APCNS acts as a semiconductor that responds to visible light, producing electron-hole pairs, while LaMnO₃ improves charge separation and redox activity; when combined, they create a heterojunction that collaboratively enhances photocatalytic efficiency by reducing recombination and broadening light absorption [33, 34].

The study also explores factors such as friction contact area, speed, and reaction vessel/beaker material on tribocatalytic performance. Additionally, it examines the influence of the synergy of photo-illumination on tribocatalytic degradation and evaluates the degradation mechanism.

## Materials and methods

### Chemicals

Lanthanum oxide (La_2_O_3_), manganese (II) acetate tetrahydrate (Mn (CH_3_COO)_2)_ (≥ 99.9% purity), hydrochloric acid (HCl), absolute ethanol (C_2_H_5_OH), sodium hydroxide (NaOH), melamine (C_3_H_6_N_6_), ammonium oxalate (C_2_H_8_N_2_O_4_), rhodamine blue, methylene blue, citric acid, nitric acid (≥ 65% purity), 1,4-benzoquinone (C_6_H_4_O_2_), isopropyl alcohol (C_4_H_10_O) was all purchased from sigma Aldrich, Italy.

### Materials synthesis

#### Lanthanum manganite (La_0.8_MnO_3_) synthesis

The La_0.8_MnO_3_ perovskite was synthesized by the citrate combustion method. The stoichiometric quantity of HNO_3_, which corresponds to La (NO_3_)_3_ and Mn (NO_3_)_2_ salts as precursors, was added to deionized water to dissolve the designated amounts of lanthanum oxide (La_2_O_3_) and manganese (II) acetate tetrahydrate. A molar ratio of 1:1 between the total amount of metal cations and citric acid monohydrate was added. To encourage the formation of gel, the solution was evaporated overnight at 120 ° C. The gel was then allowed to decompose/break down for an hour at 200 ° C in the oven. The resultant solid was ground, and it was calcined under static air for six hours at 750 °C with a ramp of 6 ° C/min.

#### Synthesis of bulk graphitic carbon nitride (B/g-C_3_N_4_)

This was synthesized according to the procedure in our previous study [33]. 10 g of melamine was weighed into a crucible and calcined in the furnace at 500 °C with a ramp rate of 5 °C/min for 4 h. After cooling, the yellow bulk material was removed and ground to powder.

#### Synthesis of acid activated porous graphitic carbon nitride sheet (APCNS)

The synthesis of Porous graphitic carbon nitride (P/g-C_3_N_4_) was reported previously in our study [33].

A conical flask (250 mL) containing 0.5 M nitric acid was combined with a known weight of P/g-C_3_N_4_ and agitated for two hours. Thereafter, the solution was centrifuged for 15 min at 6000 revolutions per minute while being washed with Millipore water, severally. The resultant product was labelled acid-activated graphitic and kept in an airtight container after being dried at 105 ° C for five hours in the oven [35].

#### Synthesis of lanthanum manganite@Acid-activated porous graphitic carbon nitride sheet (La_0_._8_MnO_3_@APCNS)

La_0_._8_MnO_3_@APCNS was synthesized by hydrothermal ultrasonication, adapted with a little modification from a study by Adewuyi and his colleagues [35]. A predetermined quantity of the synthesized APCNS was measured, then mixed with water, magnetically agitated for five hours, and then subjected to two hours of sonication. After adding a known weight of the synthesized La_0_._8_MnO_3_ and swirling the mixture for two hours, the mixture was put in an autoclave and heated to 200 ° C for six hours. The cooled mixture was then washed with deionized water and centrifuged twice for 15 min at 6000 revolutions per minute. The resultant product was dried for three hours at 105 ° C and then calcined for two hours at 450 ° C with a ramp rate of 5 ° C/min in a furnace.

### Characterization of the tribophotocatalyst

A Shimadzu 8400 S equipment was used to perform FTIR analysis on the Tribocatalyst, with measurements being made between 500 and 4000 cm^− 1^ to identify the functional groups that were present in the substance. XRD spectra were acquired using a Cu-Kα radiation source (λ = 0.154 nm), using a Bruker D8 Advance diffractometer. With EDX connected to SEM for elemental quantification at 20 kV electron acceleration voltage, SEM images were taken using a Zeiss SUPRA 40 V P microscope. A Thermo Scientific ESCALAB QXi spectrometer was utilized for the XPS study, equipped with a monochromatized Al-Kα source (h̵ ^=^ 1486.68 eV).

The Japanese-made Shimadzu model 1800 UV-Vis spectrophotometer was used to measure the amounts of RhB and MB. Using a solid sample reflectance kit and BaSO_4_ as the reference standard, the LAMBDA 1050 UV/Vis spectrophotometer (Perkin Elmer, Waltham, USA) was used to perform the UV-DRS study. The Tauc plot equation and the Kubelka-Munk function were used to determine the optical analysis, including the bandgap energy of the nanocomposite photocatalyst. specific surface area, pore diameter, average pore diameter, and total pore volume were determined on an Ultrameritics V-sorb 2802TP surface area and size analyzer.

### Tribocatalytic degradation of RhB and MB

The catalyst La_0_._8_MnO_3_@APCNS was produced and its tribocatalytic efficiency was maximized by degrading a solution of methylene blue (MB) and rhodamine blue (RhB). In a 100 mL flat bottom glass beaker (about 60 × 72 mm) with a grinding wheel coating at the bottom which provides consistent grinding and mechanical energy, 0.05 g of La_0.8_MnO_3_@APCNS interacted with a 50 mL solution of 5 mg/L of the impurities RhB and MB solution. A 3.5 cm long PTFE magnetic bar was used to magnetically swirl the mixture to create tight friction, which will cause the La_0.8_MnO_3_@APCNS particle to sink to the bottom of the beaker. The procedure was performed at 25 ° C in a completely dark environment.

Periodically, a sample of the suspension was taken, centrifuged at 5000 rpm, and then filtered through a 0.45 μm PTFE syringe filter. Using a UV visible spectrophotometer, the absorbance of the RhB and MB solutions was measured at the absorption wavelengths of 554 nm and 664 nm following the filtration process.

To ascertain their effects on the tribocatalytic degradation process, several operational variables were optimized. These variables included the number of PTFE magnetic bars (1 bar, 2 bars, and 3 bars), the material of the beaker (glass, polypropylene (PP), and Teflon), and the stirring speed (200 rpm to 700 rpm).

### Photocatalysis and tribo-photocatalysis experiment

To guarantee that the powder received enough light exposure for the photocatalysis and tribo-photocatalysis studies, a 300-W Xenon lamp (Xe Ozone free-6258) emitting in the UV to visible spectrums was employed as the illumination source. The experimental setup was the same as for the tribo-catalysis. Periodically, a sample of the suspension was taken, centrifuged at 5000 rpm, and then filtered through a 0.45 μm PTFE syringe filter. Using a UV visible spectrophotometer, the absorbance of the RhB and MB solutions was measured at the absorption wavelengths of 554 and 664 nm following the filtration process.

The percentage Tribocatalytic degradation of the RhB and MB using La_0.8_MnO_3_@APCNS was calculated using Eq. 1 below:

The amount of RhB and MB degraded was calculated using.

1 $$\% \deg radation = {{\left( {{C_0} - {C_e}} \right)} \over {{C_o}}} \times 100$$

RhB and MB equilibrium concentration is expressed in mg/L as C_e_, while RhB and MB starting concentration is expressed as C_o_.

Using the total organic carbon (TOC) analyzer, the mineralization percentage of the contaminants RhB and MB with the produced La_0.8_MnO_3_@APCNS was ascertained.

### Active specie experiment

Scavenger experiments were carried out to identify the active species that broke down RhB and MB and to comprehend the process of degradation. 50 mL of 5 mg/L RhB and MB dye solution, which are typical contaminants in our water bodies, were mixed with 0.05 g of La_0_._8_MnO_3_@APCNS. After that, the mixtures were agitated at 500 rpm while 1 mM of isopropyl alcohol (IPA), benzoquinone (BQ), and ammonium oxalate (AO), which are scavengers of positive holes and hydroxyl radicals, were added. Following the experiment, a sample aliquot was subjected to centrifugation, filtration, and UV-Visible spectrophotometer analysis.

## Results and discussion

### Characterizations

#### Fourier transform infrared spectroscopy (FTIR)

**Fig. 1:**
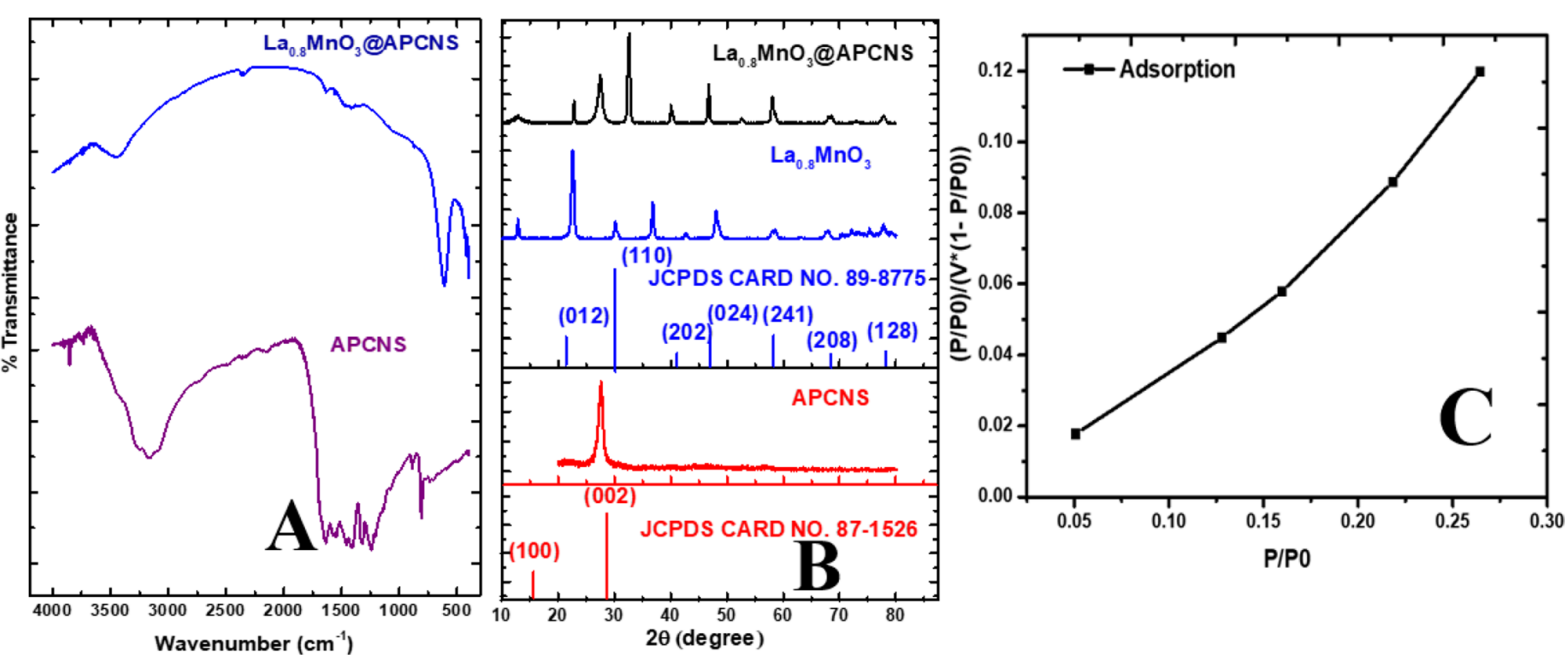
(**A**). Fourier Transformed infrared spectroscopy spectra of APCNS and La_0.8_MnO_3_@ APCNS, (**B**) X-ray Diffraction patterns of APCNS, L_0.8_MnO_3_ and La_0.8_MnO_3_@APCNS, (**C**) multipoint Brunauer–Emmett–Teller (BET) adsorption test plot of La_0.8_MnO_3_@APCNS

The FTIR spectra of La_0.8_MnO_3_@APCNS and APCNS are displayed in Fig. 1A. Between 400 and 4000 cm^-1^ was the range in which the experiment was conducted. The anticipated 4 functional groups for the materials were verified. The broad peak centered at 3400 cm^-1^ suggested the stretching vibrating mode of the N-H or O-H group indicating residual -NH or surface -OH groups on APCNS [36, 37]. The peak at 1637 cm^-1^ indicated the C = N stretching resulting from the heptazine or triazine unit in APCN. Other peaks in the range of 1230–1400 cm^-1^ revealed multiple peaks which corresponds to the existence of C-N and C = N groups of polymerized APCNS heterocycles [38], A significant peak at 805 cm^-1^ suggested the tris-S-triazine breathing mode (Adewuyi & Oderinde, 2023). The peak at 3400 cm^-1^ suggests the O-H/N-H which suggests surface hydroxyl interactions while the peak for C-N and C = N groups moved from 1637 cm to^-1^ to 1459 cm^-1^ for the La_0.8_MnO_3_@APCNS. A reduced concentration of APCNS in the sample can be the reason for the notable decrease in the strength of the C-N and C = N peaks of the APCNS in the modified sample La_0.8_MnO_3_@APCNS. The production of metal-oxygen M-O bond (Mn-O and La-O) stretching and bending vibrations was suggested by the absorption band in the 500 cm^-1^ to 700 cm^-1^ region for La_0.8_MnO_3_@APCNS [39]. Together, they form an octahedral structure of MnO_6_ [40], indicating the effective synthesis of crystalline La_0.8_MnO_3_ powder with a perovskite structure.

#### X-ray diffraction (XRD)

The crystal structure and phase composition of APCNS, La_0.8_MnO_3_, and were examined using XRD **(**Fig. 1B**).** The aromatic stacking unit forms the crystal plane (002), which is shown by the diffraction peak at 27.3^◦^ 2θ (JCPDS Card No 87-1526). The crystal planes (012), (110), (202), (024), (214), (208), and (128) corresponded to the rhombohedral structure of La_0.8_MnO_3_ perovskite (JCPDS Card No. 89-8775). The diffraction peaks of La_0.8_MnO_3_ were available at 23.0 °, 32.52 °, 41.13 °, 46.90 °, 58.56 °, 68.51 °, and 78.28 ° 2θ, respectively. The coexistence of La_0.8_MnO_3_ and APCNS in the La_0.8_MnO_3_@APCNS was confirmed by the observation of the substantial diffraction peaks corresponding to La_0.8_MnO_3_ and APCNS. This demonstrates that the La_0.8_MnO_3_@APCNS catalyst was successfully synthesized. The crystalline size of La_0_._8_MnO_3_ and APCNS in the La_0.8_MnO_3_@APCNS was calculated using the scherer equation.

2 $$d = {{K\lambda } \over {\beta {\mathop{\rm Cos}\nolimits} \theta }}$$

where D is the crystalline size, θ is the Braggs angle for the given diffraction, β is the full breadth at half maximum, and λ is the wavelength.

The greatest intensity peak of the (110) and (012) planes was used to estimate the crystalline size, which was 28.35 nm, 32 nm, and 34.20 nm, respectively.

#### Brunauer–Emmett–Teller adsorption analysis (BET)

The multipoint BET adsorption isotherm for the La_0_._8_MnO_3_@APCNS catalyst is depicted in Fig. 1C. The BET surface area measured for La_0_._8_MnO_3_@APCNS is 9.41 m²/g, accompanied by a pore diameter of 2.4 nm, indicating the presence of mesopores, as they fall within the 2–50 nm range characteristic of mesoporous materials. The pore volume of 0.22275 cm³/g demonstrates the capability of La_0_._8_MnO_3_@APCNS to effectively sequester contaminants. Despite the relatively low overall surface area, the mesoporosity of the catalyst enhances its efficacy in the removal of pollutants from water.

#### Scanning electron spectroscopy and energy dispersive X-Ray analysis (SEM&EDX)

**Fig. 2:**
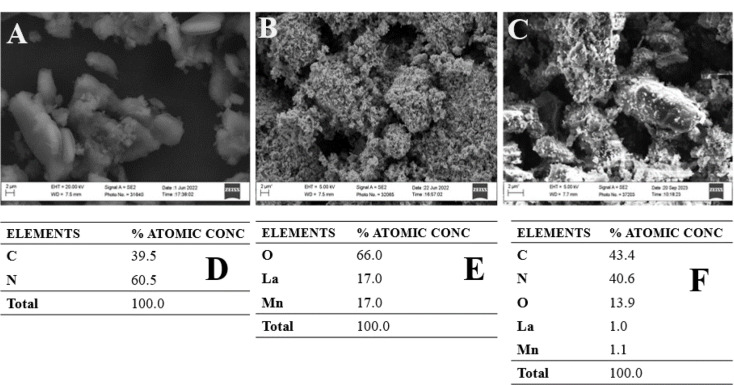
Scanning Electron Microscopy images of (**A**). APCN, (**B**). La_0.8_MnO_3_, (**C**) La_0.8_MnO_3_@APCNS, Energy Dispersive X-Ray Analysis of (**D**). APCNS, (**E**). La_0.8_MnO_3_, (F). La_0.8_MnO_3_@APCNS

The morphology of the synthesized APCNS is displayed in Fig. 2A. It consists of an ovoid-clustering network of porous forms with an apparent space in between. The morphology of La_0.8_MnO_3_ is seen in Fig. 2B, where uniform grains are dispersed over a surface that seems to be somewhat rough. Furthermore, fissures were noted. The shape of the modified La_0.8_MnO_3_@APCNS is shown in Fig. 2C. The homogenous La_0_._8_MnO_3_ grains were seen to be uniformly deposited on the APCNS surface. In addition, the changed material’s morphology revealed cavities and fissures.

It was determined by energy dispersive spectroscopy (EDX) study that La, Mn, and O make up La_0.8_MnO_3_, whereas C and N make up APCNS. Figure 2D-F illustrates the composition of La, Mn, O, N, and C in the hybrid La_0.8_MnO_3_@APCNS material. The results of the EDX analysis indicated an interaction between La_0.8_MnO_3_ and APCNS. In essence, a 0D/2D heterojunction, La_0.8_MnO_3_@APCNS, was formed. La_0.8_MnO_3_ nanoparticles and APCNS have a large contact area thanks to this heterojunction, which helps to facilitate interfacial charge transfers during the tribo-photocatalytic process.

#### X-ray photoelectron spectroscopic analysis (XPS)

**Fig. 3:**
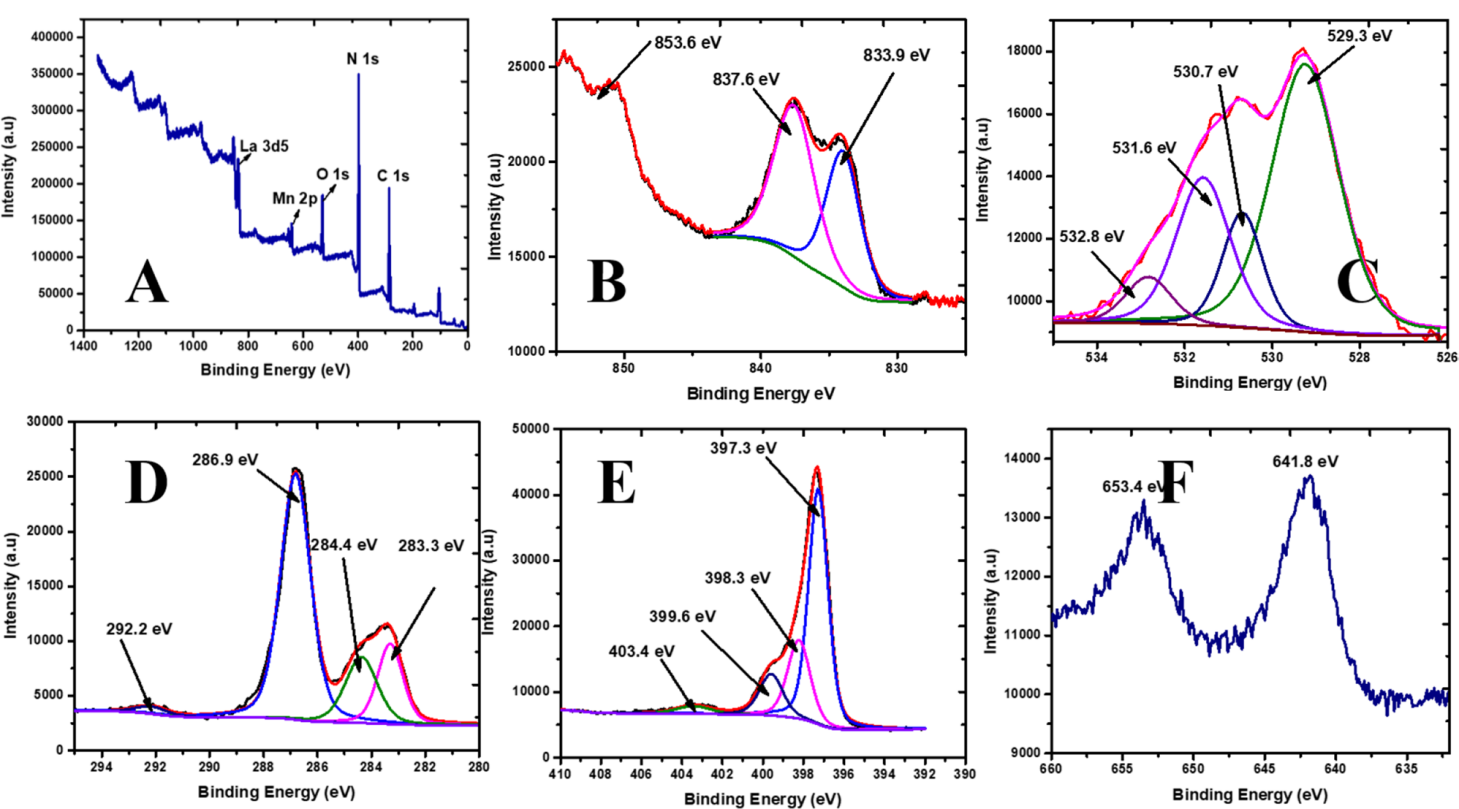
X-ray Photoelectron Spectroscopic spectra of La_0.8_MnO_3_@APCNS. (**A**) scanning survey, (**B**) La 3d photopeak, (**C**) O 1s photopeak, (**D**) C 1s photopeak, (**E**) N 1s photopeak, (**F**) Mn 2P photopeak

X-ray photoelectron spectroscopy (XPS) analysis was performed to further establish the elemental valence state and chemical composition, as well as to disclose the interfacial interaction between La_0_._8_MnO_3_ and APCNS in the as-prepared La_0.8_MnO_3_@APCNS catalyst. Figure 3 presents the acquired results. La, Mn, O, C, and N elements were present in the survey spectrum of La_0.8_MnO_3_@APCNS (Fig. 3a), which matched the chemical compositions found by energy-dispersive X-ray spectroscopy (EDX). Further analysis and peak resolution analysis were performed on the high-resolution C 1s, N 1s, La 3d, Mn 2p, and O 1s XPS spectra of La_0.8_MnO_3_@APCNS to gain a better understanding of the bonding connection between the lanthanum, manganese, oxygen, carbon, and nitrogen atoms.

The typical peaks of La 3d_5/2_ and La 3d_3/2_, respectively, were visible in the high-resolution XPS spectrum of La 3d (Fig. 3B) at 833.9, 837.6, and 853.6 eV. This indicates that La is in the chemical state of + 3 [39, 40]. Two distinct peaks, centered at 529.3 eV for the lattice oxygen (Mn O) and 531.7 eV for the hydroxyl groups that have been adsorbed on the surface, can be seen in the O 1s spectra in Fig. 3C [41, 42]. Deconvoluted C1s spectra **(**Fig. 3D**)** showed the presence of peaks at 286.8 eV, which was due to C = N, and at 283.3 eV, which was due to sp2 hybridized C atoms (C = C) [41]. While the peak at 292.2 eV was ascribed to the π-π* satellite, the peak at 284.3 eV was attributed to the sp3 hybridization of the C atom (C-C and C-H) [43]. Based on heptazine rings, bridged tertiary N atoms (N-(C)3), π-π* satellite, C-N-H, and sp2-hybridized N atoms (C-N = C), the N 1s spectrum (Fig. 3E) revealed peaks at 397.3 eV, 398.2 eV, 399.6 eV, and 403.4 eV, respectively [44, 45]. The presence of the N 1s and C 1s proved the presence of the g-C_3_N_4_.

Two distinct peaks, corresponding to Mn 2p3/2 and Mn 2p1/2, can be seen in the Mn 2p XPS spectra (Fig. 3F) at 641.8 eV and 663.4 eV, respectively. The separation between these peaks is approximately 21.6 eV, which validates the properties of La_0.8_MnO_3_ as documented in earlier research [32, 42].

**Table 1:** XPS and EDX surface composition of La_0.8_MnO_3_@APCNS

Element	XPS atomic %	EDX atomic %
N 1s	41.0	43.4
C 1s	43.8	40.6
O 1s	11.0	13.9
La 3d5	1.9	1.0
Mn 2p	2.3	1.1
Total	100.0	100.0

The surface comparison of the XPS and EDX compositions (Table 1) showed the existence of La, N, O, Mn, and C. These results also supported the effective synthesis of the La_0.8_MnO_3_@APCNS catalyst.

#### UV-DRS, Tauc plot, and XPS Valence band

**Fig. 4:**
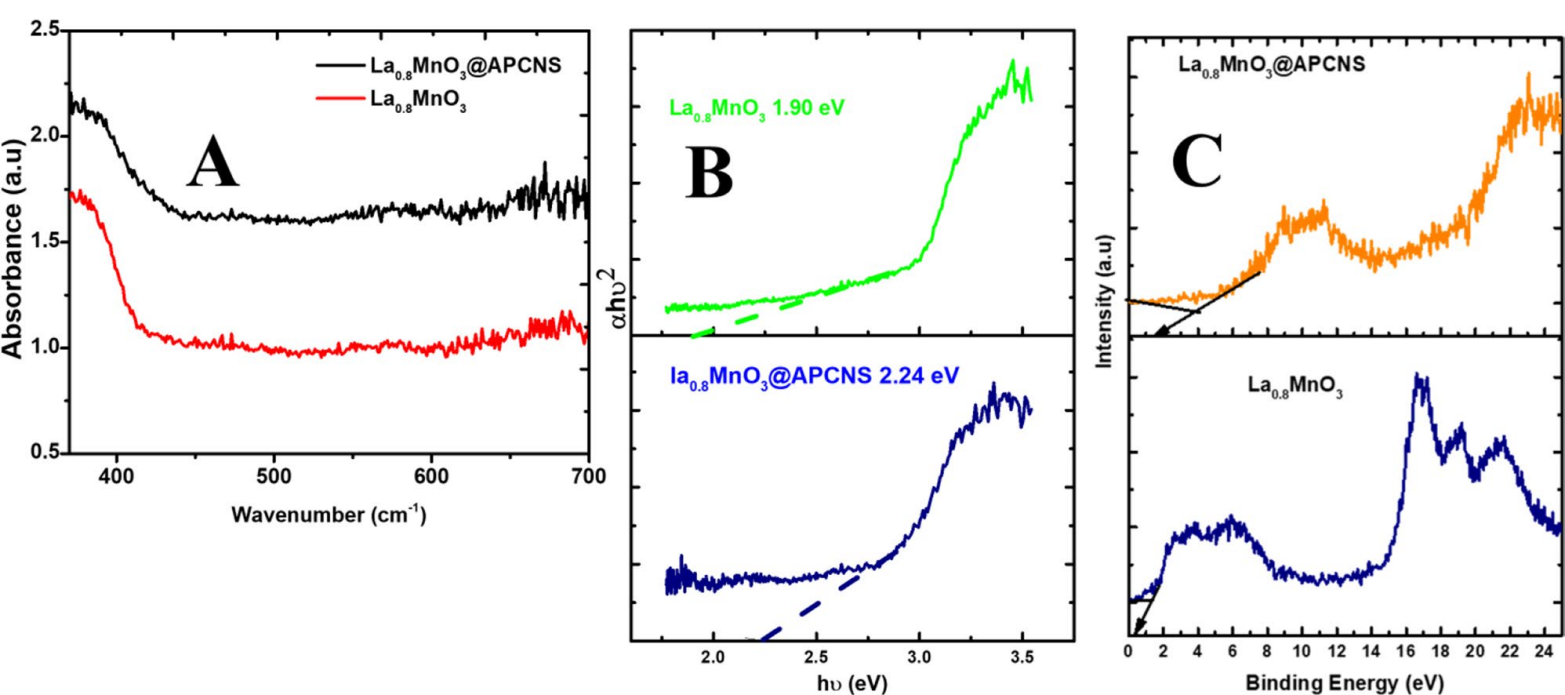
(**A**) UV Diffuse reflectance absorption. (**B**) Estimated energy band gap spectra of La_0.8_MnO_3_@APCNS (**C**). valence band XPS spectra of La_0.8_MnO_3_ and La_0.8_MnO_3_@APCNS

The UV-DRS of the catalysts La_0.8_MnO_3_ and La_0.8_MnO_3_@APCNS are shown in Fig. 4A. An absorbance band edge was observed for catalysts La_0.8_MnO_3_ and La_0.8_MnO_3_@APCNS, respectively, at approximately 550 nm and over 700 nm, indicating enhanced optical absorptions in the visible region, suggesting that the catalyst is active in the visible region (Fig. 4A).

The Kubelka-Munk equation (Eq. 3), was used to estimate the optical band gap.

3 $$\alpha hv = A\left( {hv - Eg} \right){n \over 2}$$

The semiconductor’s transition characteristics are defined by the “n” value. Direct (1) or indirect (4) can be used. Tauc’s plot, which calculates the band gap value from the extrapolated linear section of the plot of (αhv)2 vs. photon energy (hν) (eV), was used to evaluate the energy band gap (E_g_) (Fig. 4B). It is estimated that the bandgaps of La_0.8_MnO_3_@APCNS and APCNS are 1.90 eV and 2.57 eV, respectively.

In semiconductors, visible light induces the formation of electron-hole pairs in the valence band. The energized electrons then migrate to the conduction band. To comprehend the distinct valence and conduction bands of the bare La_0.8_MnO_3_ and La_0.8_MnO_3_@APCNS photocatalysts, we looked at their valence band XPS spectra, as shown in Fig. 4c. For pure La_0.8_MnO_3_ and La_0.8_MnO_3_@APCNS photocatalysts, the predicted valence band potentials are 2.38 eV and 1.4 eV, respectively.

## Degradation experiment

Before initiating any of the catalysis processes (tribocatalysis, photocatalysis, and phototribocatalysis), an adsorption-desorption equilibrium was established between La0.8MnO3@APCNS and the dyes (RhB and MB). Given that the adsorption process also contributes to the degradation of dyes, the degradation of both RhB and MB dye using 0.05 g of La0.8MnO3@APCNS through the adsorption process was approximately 15%.

### Tribocatalytic experiment

**Fig. 5:**
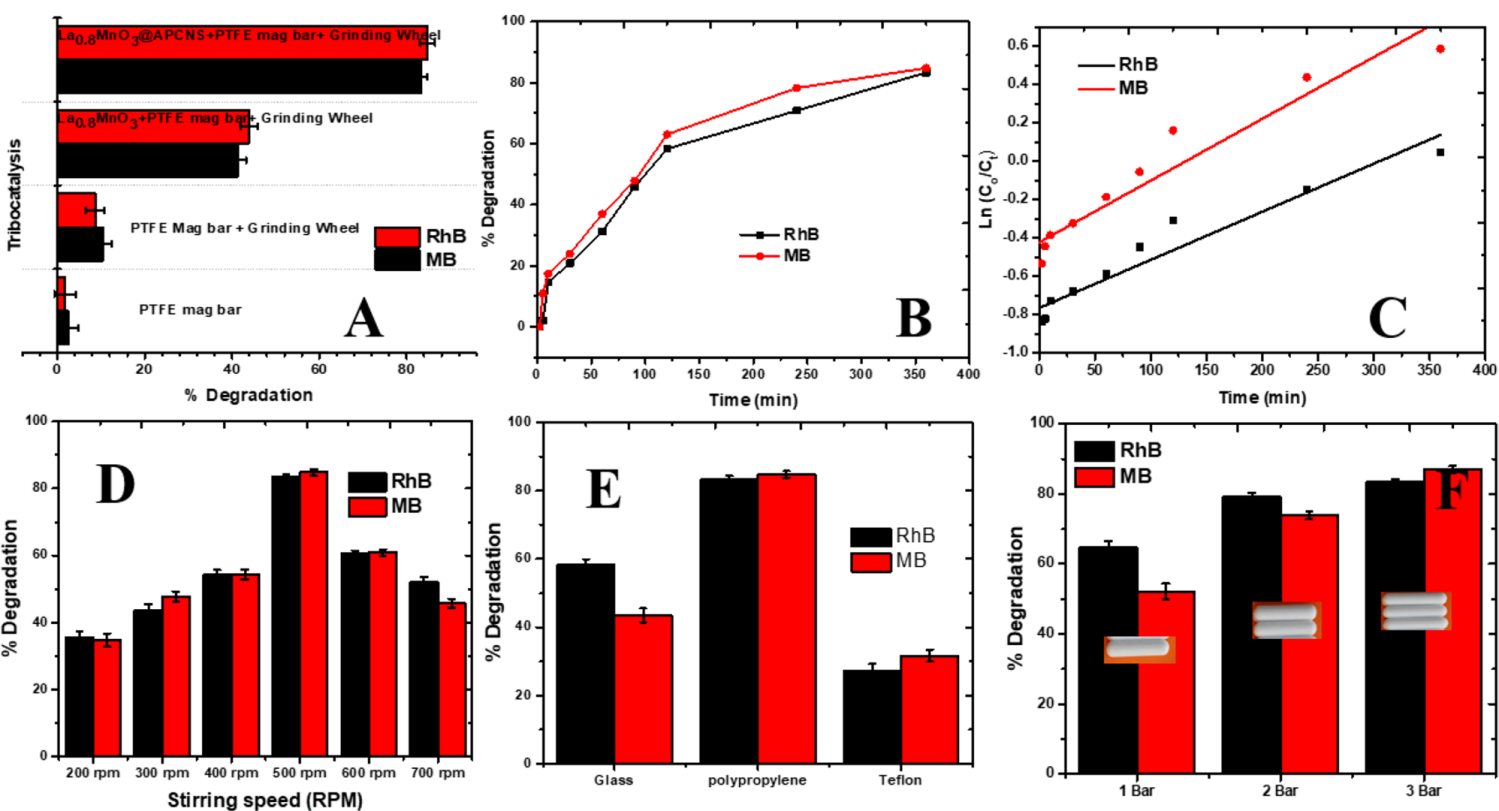
(**A**). Tribocatalytic Degradation performance of RhB and MB using PTFE magnetic bar, PTFE magnetic bar + Grinding wheel, La_0.8_MnO_3_ + PTFE magnetic bar + Grinding wheel and La_0.8_MnO_3_@APCNS + PTFE magnetic bar + Grinding wheel, Effect of (**B**) time (**c**) Pseudo-first-order kinetic model of the tribocatalytic data, (**D**) stirring speed, (**E**) different container material, (**F**) Number of PTFE magnetic bar on the tribocatalytic degradation of RhB and MB using La_0.8_MnO_3_@APCNS tribocatalyst

To evaluate the tribocatalytic activity of the synthesized materials, a series of experiments were conducted under different operational conditions. The degradation efficiency of the target contaminant RhB and MB was measured across various setups, as illustrated in Fig. 5A. The composite catalyst La_0.8_MnO₃@APCNS exhibited superior tribocatalytic performance when combined with PTFE magnetic stirring and an additional mechanical input from a grinding wheel. This setup achieved the highest degradation efficiency compared to all other tested systems.

Specifically, the La_0_._8_MnO₃@APCNS + PTFE magnetic bar + grinding wheel system achieved nearly complete pollutant removal, significantly outperforming the La_0_._8_MnO₃ + PTFE magnetic bar alone. In contrast, negligible degradation was observed in the absence of catalyst, or when only APCNS, PTFE, or the grinding wheel were used individually. These results highlight the synergistic role of catalyst design and mechanical energy input in enhancing the degradation efficiency.

The enhanced performance can be attributed to the tribocatalytic mechanism, wherein mechanical friction between PTFE magnetic bar and the catalyst surface amplified by the grinding wheel generates triboelectric charges. These charges induce separation of photo-generated electron-hole pairs in La_0.8_MnO₃, while the APCNS support facilitates rapid charge transport and inhibits recombination due to its high conductivity and porous structure.

Upon mechanical agitation, triboelectric charges are generated at the PTFE-catalyst interface. The transferred electrons react with dissolved oxygen to produce superoxide radicals (•O₂⁻), while the holes oxidize water or hydroxide ions to generate hydroxyl radicals (•OH). These reactive oxygen species (ROS) are primarily responsible for the oxidative degradation of the pollutant molecules in solution.

Thus, the incorporation of APCNS enhances the active surface area and electron mobility, while the grinding wheel intensifies triboelectric interactions and surface collisions. Together, these components facilitate efficient generation of ROS, leading to significantly enhanced degradation of kinetics under ambient conditions without the need for external light or heat sources.

As illustrated in Fig. 5B, the kinetic profile demonstrated that the degradation efficiencies for RhB and MB were 83.33% and 84.78%, respectively, after 360 min. To analyze the reaction kinetics, the tribocatalytic degradation data were fitted to a pseudo-first-order kinetic model, as described in Eq. 4 [3, 46].

4 $$\ln \left[ {{C_t}/{C_o}} \right] = - {K_1}t$$

In this context, k_1_ represents the rate constant measured in min^− 1^, C_o_ denotes the equilibrium concentration expressed in mg. L^− 1^, and C_t_ indicate the concentration of the dye, specifically RhB and MB, at a given time t, measured in minutes. The rate constants (K_*app*_) for the degradation reaction were estimated as presented in Table 2.

**Table 2:** Kinetic parameters for the tribocatalytic degradation of RhB and MB using La_0.8_MnO_3_/APCNS

Tribocatalyst (La_0.8_MnO_3_@APCNS)	k_app_ (min^− 1^)	t_1/2_ (min)	*r* ^2^
RhB MB	0.00251 0.00322	276.89 208.73	0.9662 0.9633

The highest *K*_*app*_ record was 0.00251 and 0.00322 min^− 1^, with corresponding half-lives of 276.89 and 208.73 min for RhB and MB, respectively. This data suggests that the friction-induced catalytic properties of La_0_._8_MnO_3_@APCNS facilitate a broad range of applications in the oxidative degradation of dyes. The variation in degradation rates can be attributed to the distinct oxidation-reduction properties of the primary chemical bonds present in the dyes [24].

### Effect of operational variables

A variety of optimization parameters were examined to assess the factors affecting the tribocatalytic degradation reaction of La_0_._8_MnO_3_@APCNS for Rhodamine B (RhB) and Methylene Blue (MB). These parameters included the influence of stirring speed, the type of reaction vessels employed, and the management of the contact area between the PTFE magnetic bar and La_0.8_MnO_3_@APCNS by adjusting the number of magnetic bars utilized, as illustrated in Fig. 5D-F.

The degradation efficiency of La_0.8_MnO_3_@APCNS for RhB and MB demonstrated an upward trend with increasing stirring speeds, specifically from 300 rpm to 500 rpm (Fig. 5D). This enhancement can be attributed to the accelerated mass transfer of dye molecules from the bulk solution to the catalyst surface, which in turn increased the likelihood of effective interactions among La_0.8_MnO_3_@APCNS particles. Consequently, this led to a greater transfer of electrons, thereby improving the degradation rates [47, 48]. As the rotational speed was elevated from 500 rpm to 700 rpm, a decline in efficiency was observed. This reduction can be attributed to the excessively high stirring speed, which resulted in the magnetic bar bouncing and the catalyst splattering onto the walls of the beaker during the mixing process. Consequently, this phenomenon diminished the concentration of the catalyst and reduced the frictional interaction between the PTFE magnetic bar and the La_0.8_MnO_3_@APCNS particles [46].

The impact of various beaker materials, specifically glass, Polypropylene (PP), and Teflon (PTFE), on the tribocatalytic performance of La_0.8_MnO_3_@APCNS was systematically evaluated. The degradation efficiency was observed to follow the order: PP > glass > Teflon. Notably, the degradation rate in PP beakers surpassed that of both glass and Teflon, underscoring the significant role that beaker material plays in modulating tribo-catalytic activity (Fig. 5E).

An elevated friction coefficient indicates a more substantial frictional force, which correlates with an enhanced tribo-catalytic effect of the catalyst. In the context of the tribocatalytic system, the process of magnetic stirring facilitates the interaction of catalyst particles through mutual rubbing. This interaction occurs between the PTFE-coated surface of the stirring magnet and the glass base of the reaction vessel, leading to considerable overlap of electron clouds [18, 46, 48]. Lowering the barrier between atoms enhances the transfer of electrons between materials throughout this process. Nonetheless, frictional interactions occur between the catalyst and adjacent water molecules. In the initial phases, effective charge transfer may not adequately generate variations in free radicals, resulting in constrained tribocatalytic reaction effects across various containers [49].

As the tribocatalytic reaction progresses, the plastic beaker exhibits a more significant electron transfer compared to the glass beaker, leading to a heightened production of free radicals. Consequently, the plastic beaker attained degradation levels of both dyes (83.33% and 84.78%) for 360 min, whereas the glass beaker recorded degradation rates of 58.33% and 43.47% for RhB and MB, respectively, thereby supporting the findings presented by Wu and his associates [24]. The Teflon beaker demonstrated the least effective performance, achieving a degradation efficiency of only 27.08% for RhB and 31.69% for MB. In contrast, the tribo-catalytic efficiency of La_0.8_MnO_3_@APCNS showed marked enhancement following the optimization of the arrangement of multiple magnetic rods, which increased from one to three, thereby augmenting the contact area (Fig. 5F).

### Photo-tribocatalysis study

**Fig. 6:**
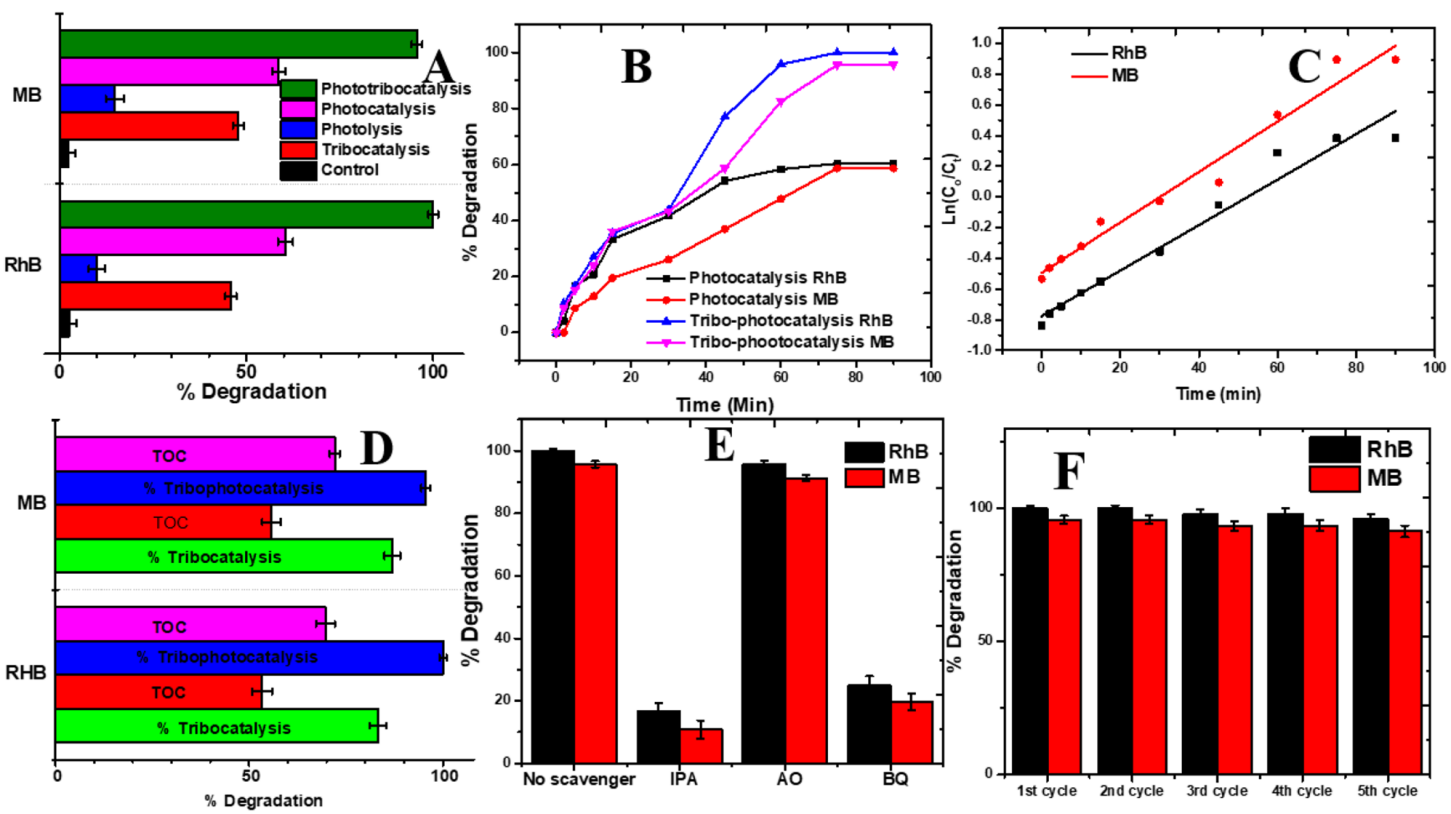
(**A**). Degradation performance of RhB and MB using just stirring (control), tribocatalysis, photolysis, photocatalysis, and tribo-photocatalysis, (**B**). kinetic profile comparing photocatalysis and tribo-photocatalysis performance (**C**) Pseudo first-order kinetic model of the tribo-photocatalysis data using La_0_._8_MnO_3_@APCNS for the degradation of RhB and MB, (**D**) showing the % degradation of RhB and MB juxtaposed against the % Total organic carbon removal (mineralization), (**E**). Reactive species scavengers experiment using La_0_._8_MnO_3_@APCNS, (**F**). reusability efficiency of La_0_._8_MnO_3_@APCNS

To examine the influence of photo-illumination on the tribocatalytic process, we conducted a series of experiments focused on the degradation of the dyes RhB and MB. The experiments included a control condition involving friction alone (stirring), as well as conditions combining friction with the addition of a catalyst (tribo-catalysis), illumination alone (photolysis), and the degradation of RhB and MB through both illumination and catalyst (photodegradation). Additionally, we assessed the combined effects of friction, illumination, and catalyst, utilizing La_0.8_MnO_3_@APCNS as the catalyst. The degradation was carried out at a stirring speed of 500 rpm over 90 min, as illustrated in Fig. 6A.

Experimental investigations revealed that the degradation efficiencies of Rhodamine B (RhB) and Methylene Blue (MB) exhibited variability under differing experimental conditions. Notably, after 90 min, the degradation efficiencies recorded were 2.5% for RhB and 2.3% for MB when subjected solely to mechanical rubbing. In contrast, the efficiencies markedly improved to 45.85% for RhB and 47.85% for MB when utilizing tribo-catalysis. Photolytic processes yielded a degradation efficiency of 10% for RhB and 14.81% for MB, whereas photocatalytic methods achieved efficiencies of 60.42% for RhB and 58.70% for MB.

Moreover, under synergistic conditions involving friction, catalyst presence, and illumination, the degradation rates after 90 min reached 100% for RhB and 95.65% for MB, as illustrated in Fig. 6B. This underscores the significant influence of combined factors on degradation efficiency. Additionally, it was observed that the interaction between La_0.8_MnO_3_@APCNS and the PTFE-coated rod during mechanical rubbing induced an electric field imbalance within the catalyst, facilitating the migration of electron-hole pairs to the catalyst surface. When exposed to xenon lamp irradiation, the catalyst generated an increased number of photogenerated electron-hole pairs, corroborated by our UV-DRS findings. The incorporation of APCNS is expected to substantially enhance the visible-light absorption capabilities of La_0.8_MnO_3_, thereby generating a greater quantity of photoinduced electron-hole pairs for photocatalytic reactions, ultimately leading to improved photocatalytic performance [32, 50].

The restricted electron transfer between La_0.8_MnO_3_@APCNS and a glass stirrer resulted in a limited separation of electron-hole pairs, which hindered their migration to the catalyst surface for degradation purposes (Gaur et al., 2024b; Jin et al., 2023).

The simultaneous effects of light exposure and mechanical friction played a crucial role in the rapid generation and separation of electrons and holes, thereby markedly improving the tribo/photo synergistic catalysis effect. This finding underscored the remarkable efficacy of the La_0.8_MnO_3_@APCNS tribocatalyst in conjunction with light-driven processes. To analyze the reaction kinetics, the tribophotocatalytic degradation data was fitted to a pseudo-first-order kinetic model, as illustrated in Fig. 6C.

**Table 3:** Kinetic parameters for the tribo-photocatalytic degradation of RhB and MB using La_0.8_MnO_3_@APCNS

Tribophotocatalyst (La_0.8_MnO_3_@APCNS)	k_app_ (min^− 1^)	t_1/2_ (min)	*r* ^2^
RhB MB	0.01483 0.01644	41.49 39.83	0.98530 0.98736

The highest *K*_*app*_ values recorded were estimated at 0.01483 and 0.01644 min^-1^, while the corresponding half-lives were 46.74 and 42.14 min for RhB and MB, respectively, as detailed in Table 3. A comparative analysis of the kinetics models for tribo-catalysis and tribo-photocatalysis revealed that the latter exhibited greater efficiency, necessitating a shorter duration for the completion of the reaction. This study investigated the effectiveness of integrating various techniques, including photolysis, tribo-catalysis, mechanical stirring, photocatalysis, and tribo-photocatalysis, for the degradation of RhB and MB, which are commonly utilized dyes. The synergistic impact of these combined techniques was assessed using the formula presented in Eq. 5, yielding a synergy value of 0.84, thereby indicating a highly effective methodology [33].

5 $$\eqalign{& synergy\,index \cr & = {{\left( {\% Deg} \right)Tribophotocatalysis} \over \matrix{\left( {\% Deg} \right)Friction + photolysis + \hfill \cr tribocatalysis + photocatalysis + tribophotocatalysis \hfill \cr} } \cr} $$

A negative synergy index value derived from Eq. 5 signifies an inhibitory effect, while a value of zero indicates a cumulative effect and a positive value reflects a synergistic process. The observed synergistic effect can be ascribed to catalyst particles generating a greater number of electron/hole pairs when subjected to light illumination and frictional forces. The capacity of La_0.8_MnO_3_@APCNS for tribo-catalysis and tribo-photocatalysis in the degradation of RhB and MB was evaluated by analyzing the Total Organic Carbon (TOC) values of the working solutions prior to and following the experimental procedure. This analysis provides valuable insights into the extent of mineralization of contaminants into CO_2_ and H_2_O. The percentage of TOC removal for tribo-catalysis was found to range from 53.4 to 55.82% for RhB and MB, while for tribo-photocatalysis, the TOC removal percentage ranged from 69.70 to 72.1% for the mineralization of RhB and MB, as illustrated in Fig. 6D.

### Regeneration and reusability study

To evaluate the capacity for cleaning, restoring, and reusing spent catalysts, a reusability study was conducted. This aspect is crucial for the practical implementation of tribo-photocatalysis in industrial applications, as it directly affects cost-effectiveness, environmental sustainability, and the overall viability of water treatment technologies. When the effectiveness of the material diminishes, regeneration facilitates the removal of absorbed contaminants, thereby restoring its efficacy for subsequent use [51].

In a recycling experiment, the recovered La_0.8_MnO_3_@APCNS was rinsed with Millipore water over 5 cycles and dried at 65 ° C. The dried regenerated catalyst obtained from the recovery was used for the subsequent round of the RhB and MB degradation experiment. It was observed that the remarkable tribo-photocatalytic performance of the La_0.8_MnO_3_@APCNS remained consistent until the 5th cycle, with a degradation efficiency exceeding 95% for RhB and over 90% for MB dyes as shown in Fig. 6E. Our findings correspond with conclusions drawn by various researchers, demonstrating the effectiveness of these methods and highlighting the potential practical and environmentally friendly nature of tribo-photocatalytic techniques for purifying water from pollutants.

### Scavenging test

The investigation into the degradation process focused on the influence of various Reactive Oxygen Species through the application of scavenger tests. Benzoquinone (BQ) served as a scavenger for superoxide, while 1mM Ammonium oxalate (AO) acted as a positive hole scavenger, and isopropyl alcohol (IPA) was utilized to scavenge hydroxyl radicals. These scavengers facilitated the elucidation of the potential degradation mechanisms of Rhodamine B (RhB) and Methylene Blue (MB) via tribo-photocatalysis on La_0.8_MnO_3_@APCNS, as illustrated **in** Fig. 6F.

The tribo-photocatalytic activity remained largely unchanged in the presence of AO, yielding efficiencies of 95.83% for RhB and 91.30% for MB. Conversely, the presence of BQ resulted in a reduction of 25% and 19.56% in efficiency for RhB and MB, respectively, while AO caused decreases of 16.67% and 10.86%. These findings indicate that hydroxyl and superoxide radicals are the predominant active species in the tribo-photocatalytic degradation of RhB and MB, ranked in the order of ˙OH >˙O_2_^−^ >h^+^. Furthermore, there exists an inverse correlation between the efficiency of tribo-photocatalytic degradation and the presence of radical scavengers; typically, the introduction of electron-trapping agents or radical scavengers leads to a decline in degradation efficiency [52, 53].

These scavengers can capture electrons or reactive radicals produced during the tribo-photocatalytic process, consequently impeding the formation of reactive species crucial for degrading dye pollutant molecules.

### Mechanism of tribo-photocatalytic degradation of RhB and MB

Previous research has established that the breakdown of organic pollutants during the tribo-catalysis process primarily occurs through mechanisms involving electron transfer and electron transition. These two mechanisms can operate concurrently, influencing the degradation process.

Our investigation reveals that the degradation process is significantly enhanced by the synergistic interaction of both tribocatalysis and photocatalysis, as illustrated in Sect. Effect of Operational Variables. The collaborative effect of these processes facilitates the degradation of RhB and MB dyes. In tribocatalysis, mechanical friction generates reactive species from water, while photocatalysis generates electron-hole pairs through exposure to light. Together, these processes contribute to the overall degradation mechanism. Mechanical stirring promotes electron transfer between atoms at the interface, thereby augmenting the electron transfer mechanism in conjunction with photocatalysis.

Regarding electron transition as a key mechanism in tribo-catalysis, mechanical stirring excites electrons from the valence band (VB) to the conduction band (CB) of the tribo-catalyst, resulting in the formation of electron-hole pairs. In this context, photocatalysis can either enhance the rate of electron excitation from the VB to the CB, as observed in the tribo-catalysis process, or it can independently support the tribo-catalysis process.

**Fig. 7:**
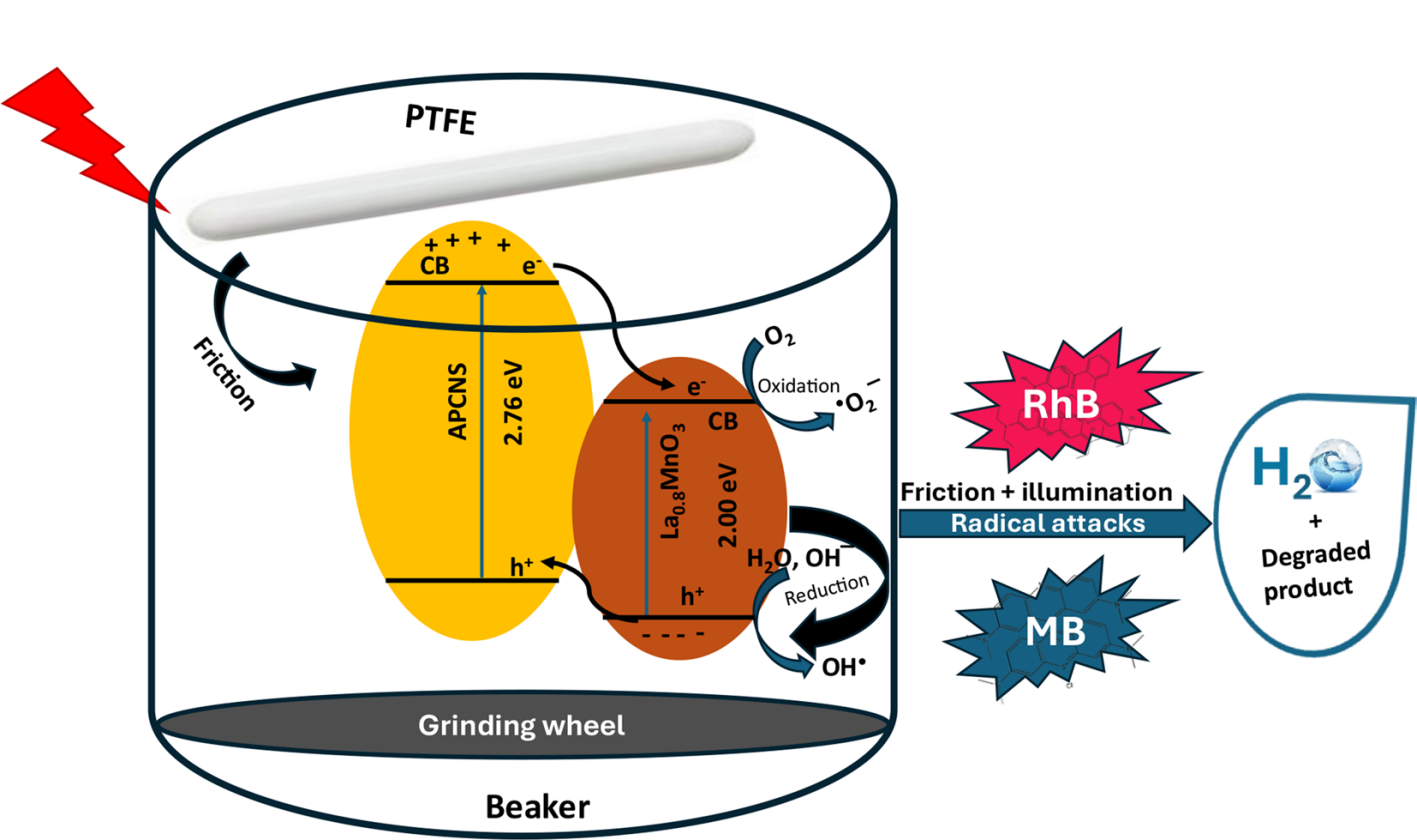
Figure showing the possible mechanism pathway for the tribo-photocatalysis of RhB and MB in water

The combined influence of both processes in the photo-tribocatalysis framework leads to the generation of a greater number of reactive species, ultimately resulting in improved degradation efficiency compared to when each process operates in isolation. In summary, photocatalysis serves to enhance the relatively lower degradation efficiency of tribocatalysis, culminating in a more effective degradation of RhB and MB dyes. The proposed schematic representation illustrates the synergistic effects of tribo-catalysis and photocatalysis for dye removal, as depicted in Fig. 7.

6 $$\text{La}\text{\_0.8}\text{MnO}\text{\_3}\text{@APCNS} + \text{hv}\,\text{to}\,\,e{\,^ - }_\text{tribo}\,e{\,^ - }_\text{photo}+ \,{\text{h}^ + }$$

7 $${{\rm{H}}_{\rm{2}}}{\rm{O + }}{{\rm{h}}^{\rm{ + }}} \to {\rm{H}}{{\rm{O}}^{\rm{ - }}}{\rm{ + }}{{\rm{H}}^{\rm{ + }}}$$

8 $${\rm{H}}{{\rm{O}}^{\rm{ - }}}{\rm{ + h}}^{\rm{ + }}{ \to }{\rm{H}}{{\rm{O}}^{\rm{ \bullet }}}$$

9 $${{\rm{O}}_{\rm{2}}}{\rm{ + }}{{\rm{e}}^{\rm{ - }}}{ \to ^{\rm{ \bullet }}}{\rm{O}}_{\rm{2}}^{\rm{ - }}$$

10 $${\,^{\rm{ \bullet }}}{\rm{O}}_{\rm{2}}^{\rm{ - }}{\rm{ + }}{{\rm{H}}^{\rm{ + }}}{ \to ^{\rm{ \bullet }}}{\rm{OOH}}$$

11 $${\,^{\rm{ \bullet }}}{\rm{OOH}}{{\rm{ + }}^{\rm{ \bullet }}}{\rm{OOH}} \to {{\rm{H}}_{\rm{2}}}{{\rm{O}}_{\rm{2}}}{\rm{ + }}{{\rm{O}}_{\rm{2}}}$$

12 $${{\rm{H}}_{\rm{2}}}{{\rm{O}}_{\rm{2}}}{\rm{ + }}{{\rm{e}}^{{\rm{ - \;\;}}}}{ \to ^{\rm{ \bullet }}}{\rm{OH + H}}{{\rm{O}}^{\rm{ \bullet }}}$$

13 $${\rm{RhB/MB Dye }}{{\rm{ + }}^{\rm{ \bullet }}}{\rm{OH}}{{\rm{/}}^{\rm{ \bullet }}}{{\rm{O}}_{\rm{2}}} \to {\rm{Degradation Byproducts}}$$

Comparison of our catalyst with previously reported materials for the degradation of RhB and MB, as shown in Table 4, and La_0_._8_MnO_3_/APCNS performance was observed to be outstanding, confirming it to be a very efficient material for the degradation of both dyes RhB and MB.

**Table 4:** A table showing the comparison of the degradation of RhB and MB in water using other reported catalysts

Catalyst	Contaminant	Degradation efficiency	References
CoFe_2_O_4_@Ag	RhB	97.42	[54]
9% g-C_3_N_4_/ZrO_2_	MB	99	[55]
3D PUF/rGo/ZnO	MB	70	[56]
MgFe_2_O_4_-TiO_2_	MB	99.53	[57]
ZnO-GO-Fe_3_O_4_	MB	97	[58]
g-C_3_N_4_	RhB	98	[59]
ZnCl_2_-activated carrot waste AC	RhB	100	[60]
Magnetic N-C dots	MB RhB	92.76 83.05	[61]
C-TiO_2_/g-C_3_N_4_	RhB	96.6	[62]
La_0_._8_MnO_3_@APCNS	RhB MB	100 95.65	This study

## Conclusion

There is a growing need to explore cost-effective techniques for the degradation of organic contaminants, hence tribocatalysis was explored, but there is a major disadvantage of longer reaction time. In a bid to overcome this shortcoming, the synergistic effect of combining tribocatalysis and photocatalysis processes was explored in our study. This is poised to serve as an important step towards the improvement of the performance of the individual process.

La_0.8_MnO_3_@APCNS, a flexoelectric material, was successfully synthesized and explored for the degradation of RhB and MB. In the tribocatalysis process where, the PTFE magnetic bar, grinding wheel, and La_0.8_MnO_3_@APCNS catalys were used, the degradation efficiency was observed to be 83.33% and 84.78% in 360 min, while for the tribo-photocatalysis the degradation efficiency was observed to be 100% and 95.65% in 90 min for RhB and MB respectively.

The tribo-photocatalytic process proved to be the best with a shorter reaction time and the synergy index was calculated to be 0.84, indicating a highly effective approach. The degradation in the tribocatalysis process was due to electron transfer caused by atom transfer between La_0.8_MnO_3_@APCNS and PTFE magnetic bar interface whereas the electron-hole pair was produced in the photocatalysis process resulting in the production of reactive species. Hence the combined effect of both processes resulted in enhanced degradation as the photocatalysis process aids the -tribo-photocatalysis process. It was observed that the remarkable tribo-photocatalytic performance of the La_0.8_MnO_3_@APCNS remained consistent until the 5th cycle, with a degradation efficiency exceeding 95% for RhB and over 90% for MB dyes, demonstrating the effectiveness of these methods and highlighting the potential practical and environmentally friendly nature of tribo-photocatalytic techniques for purifying water from pollutants. Tribophotocatalysis using La_0.8_MnO_3_@APCNS has the potential to transform dye degradation processes. However, current research is limited by a lack of understanding of the underlying mechanisms, issues with material stability, and an absence of testing in real-world applications. Future studies should emphasize the development of smart hybrid systems, provide deeper mechanistic insights, and conduct validations in real-world environments to connect laboratory innovations with scalable environmental impact.

## Data Availability

The datasets used and/or analyzed during the current study are available from the corresponding author upon reasonable request.
